# Tea Polyphenol Attenuates Oxidative Stress-Induced Degeneration of Intervertebral Discs by Regulating the Keap1/Nrf2/ARE Pathway

**DOI:** 10.1155/2021/6684147

**Published:** 2021-01-07

**Authors:** Dawei Song, Jun Ge, Yingjie Wang, Qi Yan, Cenhao Wu, Hao Yu, Ming Yang, Huilin Yang, Jun Zou

**Affiliations:** Department of Orthopaedic Surgery, The First Affiliated Hospital of Soochow University, Suzhou, Jiangsu 215006, China

## Abstract

**Objective:**

Intervertebral disc degeneration (IDD) and low back pain caused by IDD have attracted public attention owing to their extremely high incidence and disability rate. Oxidative stress is a major cause of IDD. Tea polyphenols (TP) are natural-derived antioxidants extracted from tea leaves. This study explored the protective role of TP on the nucleus pulposus cells (NPCs) of intervertebral discs and their underlying mechanism.

**Methods:**

An *in vitro* model of H_2_O_2_-induced degeneration of NPCs was established. RT-qPCR and western blotting were used to detect the mRNA and protein expression of the targets. An *in vivo* model of IDD was established via acupuncture of the intervertebral disc. Radiological imaging and histological staining were performed to evaluate the protective role of TP.

**Results:**

H_2_O_2_ contributed to NPC degeneration by inducing high levels of oxidative stress. TP treatment effectively increased the expression of nucleus pulposus matrix-associated genes and reduced the expression of degeneration factors. Further mechanistic studies showed that TP delayed H_2_O_2_-mediated NPC degeneration by activating the Keap1/Nrf2/ARE pathway*. In vivo* experiments showed that TP delayed the degeneration of NPCs in rats through the Keap1/Nrf2/ARE pathway.

**Conclusion:**

Our study confirmed that TP activates the Keap1/Nrf2/ARE pathway to exert an antioxidative stress role, ultimately delaying the degeneration of intervertebral discs.

## 1. Introduction

Chronic low back pain induced by intervertebral disc degeneration (IDD) is limited to symptomatic treatment such as conservative medication or surgical intervention [1]. Commonly used drugs include nonsteroidal anti-inflammatory drugs, skeletal muscle relaxants, and opioids. However, drug treatment only temporarily relieves the symptoms and cannot fundamentally reverse the progression of disc degeneration in patients. Surgical removal of the intervertebral disc is usually used for patients with severe symptoms. Although effectively alleviating pain, surgical intervention is risky and expensive [2]. Moreover, surgical operation destroys the normal physiological structure of the spine and causes degeneration of adjacent segments, which cannot fundamentally reduce the incidence of disc degeneration. Neither pharmaceutical nor surgical treatments can delay IDD progression. Therefore, it is clinically meaningful to elucidate the pathogenesis of IDD and develop a novel therapy to prevent disc degeneration.

It is well known that a variety of factors contribute to IDD, including inflammation and biomechanical stimulation. Recent studies have shown that oxidative stress plays an important role in the pathological mechanism of diverse diseases, including the degeneration of intervertebral discs [3, 4]. Mitochondrial dysfunction induced by oxidative stress is related to the pathogenesis of disc degeneration [5, 6] because mitochondria are the main source of reactive oxygen species (ROS) in cells. On the one hand, a large amount of ROS is produced during oxidative stress; on the other hand, the scavenging function of ROS in the body's antioxidant system is attenuated, disrupting the dynamic balance between ROS production and elimination [7]. ROS are an important mediator of the cell signaling network in the intervertebral disc cells, regulating matrix metabolism, proinflammatory reactions, apoptotic cell death, autophagy, and aging [8]. Oxidative stress not only enhances the peripheral matrix degradation and inflammatory response in intervertebral disc cells but also attenuates cell number and cellular function in the intervertebral disc microenvironment [9]. In addition, ROS also modify the peripheral matrix protein, accelerate the oxidative damage of the extracellular matrix, and damage the mechanical function of the intervertebral disc [8]. Therefore, targeting oxidative stress may provide a new perspective for IDD treatment. A variety of antioxidants are considered effective drugs for the treatment of IDD [10, 11].

Given their plant origin, tea polyphenols (TP) extracted from tea leaves are a safer antioxidant than synthetic counterparts. TP are considered to be chain-breaking antioxidants, based on their potential for hydrogen atom transfer or single electron transfer reactions (SETs). Many studies have confirmed the antioxidant effects of TP, including prevention of oxidative stress and regulation of carcinogen metabolism [12]. In kidney diseases, damage, and toxic situations, e.g., excessive arginine supply, strong oxidative radicals, renal toxin, and diabetic nephropathy, TP play a beneficial role against pathological reactions related to oxidative stress [13]; TP also enhance the antioxidant activity of liver cells, making them a potential treatment of liver cancer [14]. Breast cancer cells pretreated with TP become more sensitive to methotrexate because of the reduction of intracellular ROS. TP also improve the cognitive impairment caused by chronic cerebral hypoperfusion by regulating oxidative stress reactions [15].

There is limited research on the effect of TP on the intervertebral disc, especially in the nucleus pulposus tissues. Given the unique antioxidative stress properties of TP, our study was aimed at exploring their protective roles in the nucleus pulposus cells (NPCs) from the intervertebral disc. The study verified the effect of TP on the nucleus pulposus using *in vitro* and *in vivo* assays.

## 2. Materials and Methods

### 2.1. Cell Line, Reagents, and Instruments

**Scheme 1:**
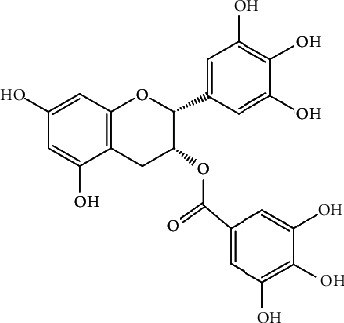
Chemical formula of the tea polyphenols.

Fetal bovine serum, Dulbecco's modification of Eagle's medium (DMEM), Trizol reagent, a reverse transcription kit, and a SYBR Green Real-Time PCR Master Mix kit were purchased from Thermo Fisher Scientific (San Jose, CA, USA). RAPI protein lysates and a BCA kit were purchased from Invitrogen (Carlsbad, CA, USA). Primary antibodies against collagen II, aggrecan, SOX-9, collagen X, MMP3, iNOS, NOX4, SOD2, LDH, T-AOC, NRF2, KEAP1, and ARE as well as the corresponding secondary antibodies were purchased from Wuhan Amictech Technology Co., Ltd. (Wuhan, Hubei, China). A CCK-8 kit was purchased from Shanghai Yisheng Biotechnology Co., Ltd. (Shanghai, China). A microplate reader, a fluorescence quantitative PCR amplifier, and an electrophoresis and gel imaging system were purchased from Thermo Fisher Scientific (San Jose, CA, USA); an ultracentrifuge and electrophoresis tank were purchased from Beijing Liuyi Biotechnology Co., Ltd. (Beijing, China). TP (epigallocatechin-3-gallate (ECGC) Scheme 1) was purchased from Hangzhou Yibeijia Tea Technology Co. Ltd.

### 2.2. Cell Culturing

Cells (nucleus pulposus cell lines, Crisprbio, Beijing, China) were cultured in DMEM containing 10% fetal bovine serum, 100 U/mL penicillin, and 0.1 mg/mL streptomycin at 37°C with 5% CO_2_. NPCs in the log phase were digested with trypsin, seeded in a 6-well plate (10^4^ cells/well), and incubated at 37°C for 24 h for later use.

### 2.3. NPC Viability Assay

NPC cells were seeded in a 96-well plate at a density of 5 × 10^3^ cells/well and cultured at 37°C in an incubator for 24 h. CCK-8 solution (10 *μ*L) was then added to each well. After incubation at 37°C for 2 h, optical absorbance was detected on a microplate reader, with at least three replicates.

### 2.4. Quantitative Real-Time PCR (RT-qPCR)

Total RNA was extracted from tissues and cells using Trizol reagent. The RNA concentration was measured using agarose gel electrophoresis and reverse transcribed into cDNA. The mRNA levels of collagen II, aggrecan, SOX-9, collagen X, MMP3, iNOS, NOX4, SOD2, LDH, T-AOC, NRF2, KEAP1, and ARE were detected using a SYBR Green kit, and U6 served as an internal reference. Primers for gene amplification were designed using the Primer Premier 5.0 software. PCR reactions were performed at 95°C for 5 min, followed by denaturation at 94°C for 30 s and annealing at 60°C for 30 s, with a total of 45 cycles, in a final volume of 20 *μ*L, including 2 *μ*L cDNA, 10 *μ*L SYBR Green Mix, 0.5 *μ*L upstream and 0.5 *μ*L downstream primers (10 *μ*mol/L each), and 7 *μ*L dH_2_O. Relative gene levels were calculated by the 2-*ΔΔ*Ct method. The experiment was repeated three times independently. Prime sequence could be found in Table 1.

### 2.5. Western Blotting

Total proteins were extracted with RIPA lysis buffer, and their concentration and purity were measured with a BCA kit. Proteins were subjected to 10% SDS-PAGE at 80-120 V and transferred to polyvinylidene fluoride (PVDF) membranes at 100 mV for 1 h. The membranes were blocked in 5% skimmed milk powder for 1 h and incubated with the primary antibody (1 : 1,000) overnight at 4°C. The next day, the membranes were washed three times with Tris-Buffered Saline Tween-20 (TBST) for 5 min and incubated with the secondary antibody (1 : 5,000) at room temperature for 1 h. After washing the membranes three times with TBST, protein signals were developed using an ECL kit; the images were captured using a gel imaging system and quantitatively analyzed using ImageJ software. The experiment was repeated three times independently.

### 2.6. Animal Model

In total, 18 specific pathogen-free (SPF) male rats, weighing 400 ± 20 g, were purchased from Nanjing Pengsheng Biotechnology Development Co., Ltd. (Nanjing, Jiangsu, China). MRI and X-ray examination confirmed no congenital caudal disc deformity or degeneration. The rats were randomly divided into three groups using the digital table method: the degeneration group (normal saline group), the TP group (100 nM), and the TP+NRF2 siRNA group, with six rats in each group. The rats were weighed and intraperitoneally injected with ketamine hydrochloride (50 mg/kg) and piperazine hydrochloride (5 mg/kg). After anesthesia, the rats were fixed and disinfected, and the co8/9 vertebral space was penetrated to the opposite side completely and rotated 360° for 30 s using an 18G needle with X-ray guidance. Immediately, 2 *μ*L TP saline (100 *μ*M), TP (100 *μ*M)+Nrf2 siRNA(100 *μ*M, Santa Cruz Biotech, Santa Cruz, CA) saline, and an equal amount of normal saline were injected into the intervertebral discs. Following surgery, rats had free access to food and water, and urinary retention and infection were closely monitored. All the experiments, conducted at the First Affiliated Hospital of Soochow University, were approved by the Ethics Committee and strictly abide by the “Declaration of Helsinki” (1964) and “Guidelines for Ethical Review of Laboratory Animal Welfare” (GB/T 35892-2018, China).

### 2.7. MRI Scan

The tails of the rats were exposed to MRI scans at weeks 1, 2, and 4 after surgery. First, rats were anesthetized by intraperitoneal injection with ketamine hydrochloride (50 mg/kg) and piperazine hydrochloride (5 mg/kg). The intervertebral disc signal was then obtained on a 1.5 T magnetic resonance scanner (Philips Eclipse, Cleveland, OH, USA), with the following parameters of the T2-weighted sagittal plane: TR/TE, 3,500/102 ms; FOV, 15.0; thickness, 3 mm; and interval, 0 mm. The degree of disc degeneration was assessed by signal intensity on a T2-weighted image (T2WI) of the intervertebral disc. The degree of IDD was evaluated based on T2WI signal intensity, including quantitative analysis of T2 phase signal using ImageJ, and disc grading by the modified Pfirrmann system (Table 2). The grading was performed independently by two experienced spinal surgeons. When the grades diverged, a third and more senior spinal surgeon was asked to evaluate the results, and three spinal surgeons negotiated and gave the final decision.

### 2.8. Histology

Following radiological examination, the rats were euthanized. The Co8/9 intervertebral discs were removed, fixed with 10% neutral formaldehyde at room temperature for 24 h, decalcified with 15% EDTA for 4 weeks, embedded, and sectioned. The sectissons were deparaffinized twice in xylene for 5 min, dehydrated in graded ethanol (75%, 1 min; 80%, 1 min; 95%, 1 min; and 100%, 2 min), and stained with hematoxylin for 5 min and with eosin for 2 min. Morphological changes of discs were observed under a light microscope and scored according to the classification criteria described in Table 3.

### 2.9. Immunohistochemical Staining

Immunohistochemistry was used to detect the expression of type II collagen, type X collagen, and SOD2. The sections were deparaffinized with xylene, dehydrated with graded ethanol, and incubated at 37°C with 3% H_2_O_2_ for 10 min. After being washed three times in PBS, the sections were boiled in 0.01 M citrate buffer (95°C, 15-20 min) and blocked in goat serum at 37°C for 10 min. The sections were then incubated with the primary antibody at 4°C overnight and the biotin-labeled secondary antibody (BioWorld, Visalia, CA, USA) at 37°C for 30 min. The sections were counterstained with hematoxylin and observed under a light microscope.

### 2.10. Statistical Analysis

The SPSS 18.0 software was used for statistical analysis. Differences between the two groups were analyzed using the *t*-test, and differences between multiple groups were analyzed using one-way analysis of variance. GraphPad Prism 8.2 was used to draw relevant figures. Values of *P* < 0.05 indicated statistical significance.

## 3. Results

### 3.1. H_2_O_2_-Induced NPC Degeneration

**Figure 1:**
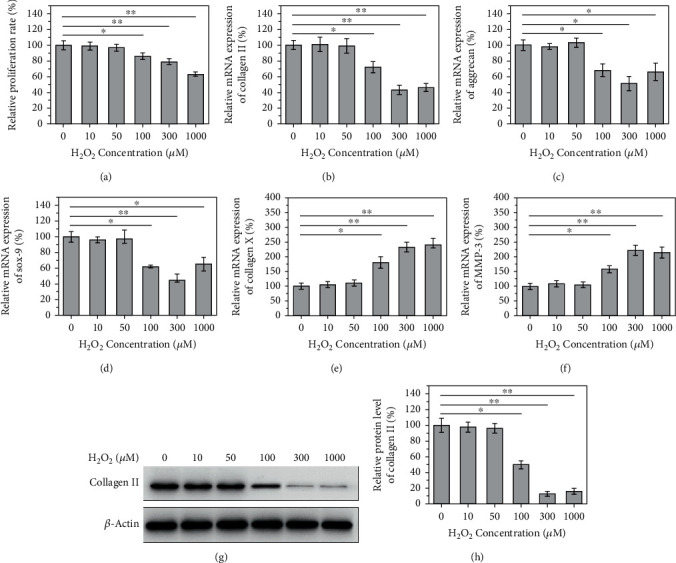
100 *μ*M H_2_O_2_ induces the degeneration of nucleus pulposus cells. When the concentration of H_2_O_2_ was more than 100 *μ*m, (a) the proliferation rate of cells decreased significantly. The relative mRNA expression of (b) collagen II, (c) aggrecan, and (d) Sox-9 decreased significantly, while that of (e) collagen X and (f) MMP-3 increased significantly. (g, h) And the relative protein level of collagen II decreased significantly (*P* < 0.05).

A CCK-8 assay revealed that H_2_O_2_ at 100 *μ*M had no obvious effects on NPC viability, whereas higher doses of gradually decreased it (Figure 1(a), *P* < 0.05). Thus, H_2_O_2_ at 100 *μ*M was chosen for further study. RT-qPCR results showed that mRNA levels of collagen II, aggrecan, and SOX-9 were significantly decreased (Figures 1(b)–1(d)), whereas collagen X and MMP3 levels were elevated (Figures 1(e) and 1(f)), following treatment with H_2_O_2_ at 100 *μ*M or higher doses (*P* < 0.05). Western blotting identified a decrease in the protein expression of collagen II in NPCs treated with H_2_O_2_ at 100 *μ*M or higher (Figures 1(g) and 1(h), *P* < 0.05).

### 3.2. H_2_O_2_ Induces NPC Degeneration via High Oxidative Stress

**Figure 2:**
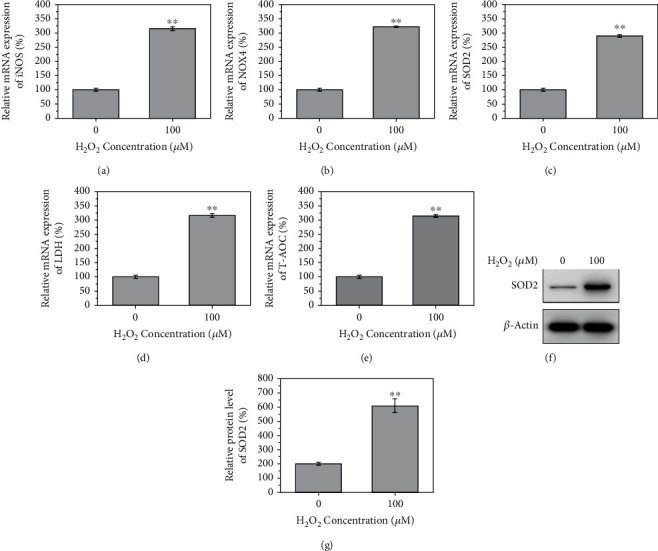
100 *μ*M H_2_O_2_ caused oxidative stress in nucleus pulposus cells. (a–e) The relative mRNA of iNOS, NOX4, SOD2, LDH, and T-AOC significantly increased after the treatment of 100 *μ*M H_2_O_2_. (f, g) The relative protein level of SOD2 increased significantly after the treatment of 100 *μ*M H_2_O_2_.

The RT-qPCR data unveiled that mRNA levels of iNOS, NOX4, SOD2, LDH, and T-AOC were significantly increased in NPCs exposed to 100 *μ*M H_2_O_2_ (Figures 2(a)–2(e), *P* < 0.05). In addition, western blotting showed that SOD2 protein levels were dramatically elevated following treatment with 100 *μ*M H_2_O_2_ (Figures 2(f) and 2(g), *P* < 0.05).

### 3.3. TP Delays H_2_O_2_-Induced NPC Degeneration

**Figure 3:**
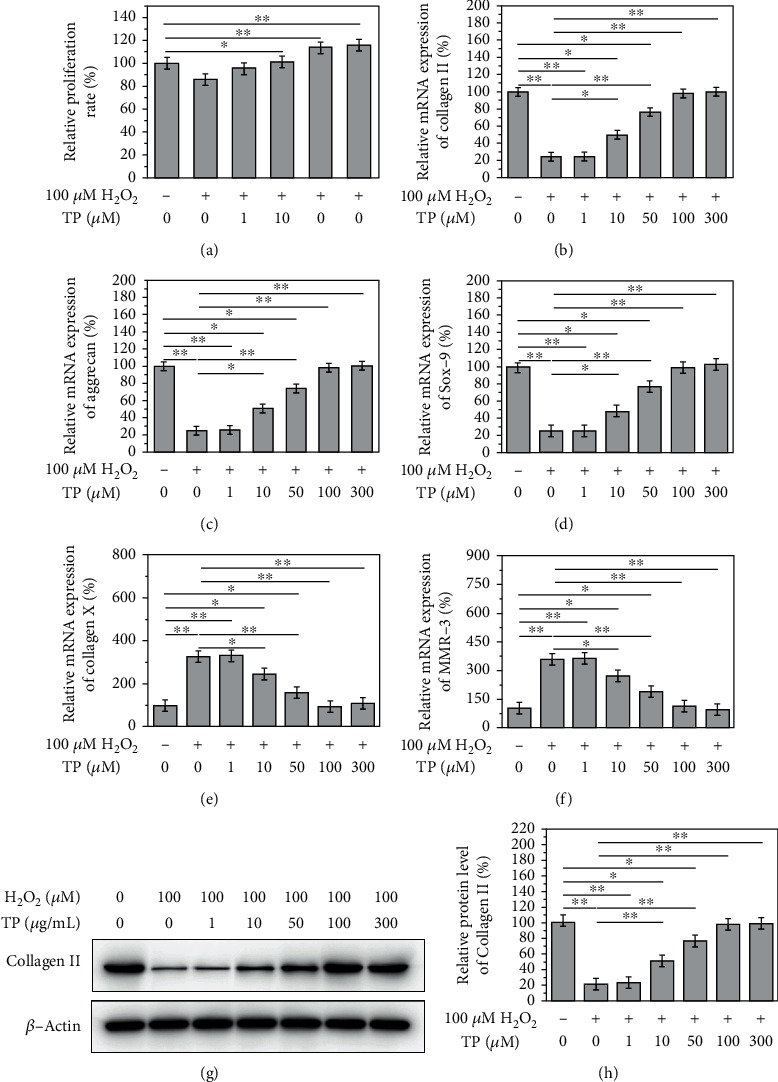
TP alleviated the degeneration of nucleus pulposus cells caused by H_2_O_2_ in a dose-dependent manner. After the treatment of TP, (a) the proliferation rate of cells came back to normal. The relative mRNA expression of (b) collagen II, (c) aggrecan, and (d) Sox-9 increased significantly, while that of (e) collagen X and (f) MMP-3 decreased significantly. (g, h) And the relative protein level of collagen II increased significantly (*P* < 0.05).

Data from the CCK-8 assay suggested that TP at low doses had no obvious effects on H_2_O_2_-treated NPCs. However, TP (>50 *μ*g/mL) clearly promoted H_2_O_2_-treated NPC cell viability (Figures 3(a), *P* < 0.05). RT-qPCR assay showed that treatment with TP significantly elevated mRNA levels of collagen II, aggrecan, and SOX-9 and decreased the levels of collagen X and MMP3 in 100 *μ*M H_2_O_2_ treated NPCs (Figures 3(b)–3(f), *P* < 0.05). Moreover, western blotting analysis showed that TP could increase protein expression of collagen II in NPCs treated with H_2_O_2_ (100 *μ*M) (Figures 3(g) and 3(h), *P* < 0.05).

### 3.4. TP Effectively Reduces H_2_O_2_-Mediated High Oxidative Stress in NPCs

**Figure 4:**
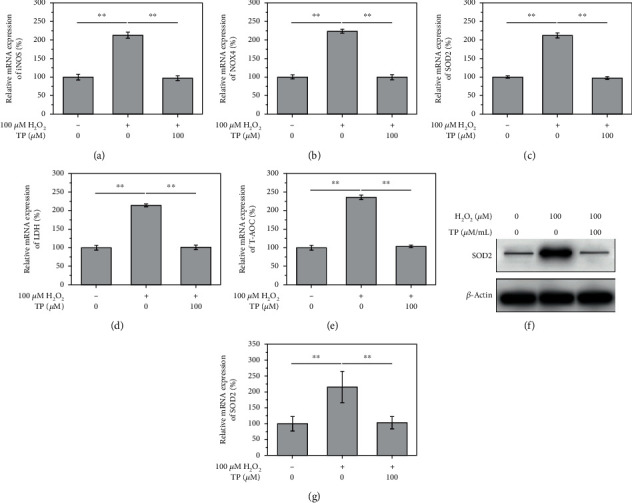
TP alleviated the oxidative stress in nucleus pulposus cells caused by H_2_O_2_. (a–e) The relative mRNA of iNOS, NOX4, SOD2, LDH, and T-AOC came back to normal after the treatment of both H_2_O_2_ and TP. (f, g) The relative protein level of SOD2 came back to normal after the treatment of both H_2_O_2_ and TP.

RT-qPCR results showed that TP (100 *μ*g/mL) significantly inhibited the mRNA expression of iNOS, NOX4, SOD2, LDH, and T-AOC in NPCs treated with 100 *μ*M H_2_O_2_ (Figures 4(a)–4(e), *P* < 0.05). In addition, TP (100 *μ*g/mL) treatment significantly suppressed protein expression of SOD2 in NPCs treated with 100 *μ*M H_2_O_2_ (Figures 4(g) and 4(h)).

### 3.5. TP Activates the Keap1/Nrf2/ARE Pathway to Delay H_2_O_2_-Induced NPC Degeneration

**Figure 5:**
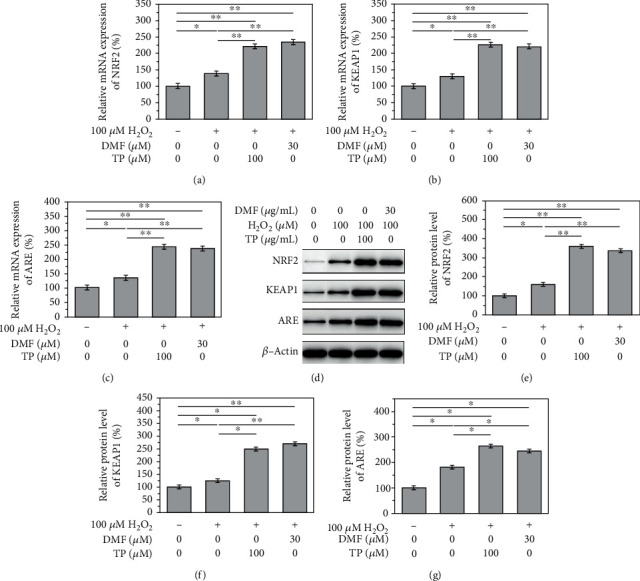
TP exert its function via the Nrf2/KEAP1/ARE pathway. After the TP treatment, the relative mRNA expression of (a) Nrf2, (b) KEAP1, and (c) ARE significantly increased, (d–g) so does the protein level of Nrf2, KEAP1, and ARE (*P* < 0.05).

RT-qPCR and western blotting showed that TP (100 *μ*g/mL) increased the mRNA and protein levels of NRF2, KEAP1, and ARE (*P* < 0.05), similar to the effects of dimethylformamide (DMF), an activator of the Keap1/Nrf2/ARE pathway (Figures 5(a)–5(c)). H_2_O_2_ treatment alone slightly promoted the expression of NRF2, KEAP1, and ARE. Combined treatment with TP (100 *μ*g/mL) and H_2_O_2_ significantly promoted the mRNA and protein levels of NRF2, KEAP1, and ARE (*P* < 0.05). Moreover, DMF (30 *μ*g/mL) combined with TP (100 *μ*g/mL) further increased the mRNA and protein expression of NRF2, KEAP1, and ARE in NPCs treated with 100 *μ*M H_2_O_2_ (*P* < 0.05) (Figures 5(d)–5(g)).

### 3.6. TP Delays NPC Degeneration via the Keap1/Nrf2/ARE Pathway *In Vivo*

#### 3.6.1. MRI Examination and Pfirrmann Grading

**Figure 6:**
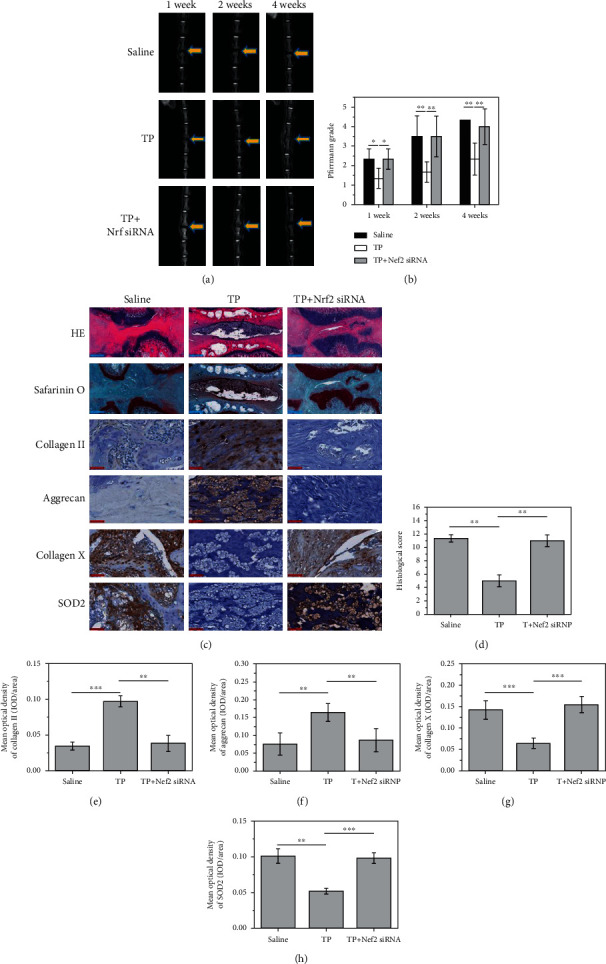
TP delays the intervertebral disc degeneration in vivo via the Nrf2/KEAP/ARE pathway. (a) MRI test of rat caudal discs reveals the protective role of TP in intervertebral disc degeneration. (b) Pfirrmann grade showed the significant protective role of TP. (c) Histological analysis of caudal discs. (d) Histological grading test. (e–h) Mean optical density of collagen II, aggrecan, collagen X, and SOD2 (*P* < 0.05). Blue scale bar = 500 *μ*m; red scale bar = 50 *μ*m.

MRI examination showed that TP treatment improved disc degeneration. Although rats in the saline degeneration group maintained the original height of the intervertebral space in the first week, the disc signal significantly decreased; however, TP-treated rats showed a high disc signal level in the first week. Although the signal was somewhat indistinct and dispersed compared to the signal observed in the control group, the overall situation was better than that of the degeneration group (saline). In the MRIs performed at the second and fourth weeks, it was observed that the discs in the degeneration group became “dark discs” with an obvious decreased height of the intervertebral space. Conversely, the discs in the TP group showed an obvious improvement and retained high signals; the signal of the disc did not go dark sharply, although the height of the intervertebral space continued to decrease. The addition of NRF2 siRNA to the TP solution decreased NRF2 mRNA levels, which accelerated the degeneration of the intervertebral disc (Figure 6(a)).

Quantitative and qualitative analyses further confirmed these results. Gray value analysis showed that TP effectively reduced the gray levels of the intervertebral disc signal, resulting in a high signal state (*P* < 0.05). The Pfirrmann scores revealed significant differences between the TP and degeneration groups in the first week (*P* < 0.05). At the fourth week, such differences became more obvious among the TP, degeneration, and TP+Nrf2 siRNA groups (*P* < 0.05). Compared with the degeneration group, TP treatment alleviated disc degeneration *in vivo* (Figure 6(b)).

### 3.7. HE Staining and Histology Analysis

After four weeks of treatment, HE staining in the degeneration group showed that the volume of the intervertebral nucleus pulposus and the number of cartilage cells were reduced. In some sections, the nucleus pulposus tissue completely disappeared, and the fibrous annulus appeared to be twisted and damaged. Similar results were observed in the TP+NRF2 siRNA group. However, treatment with TP for four weeks significantly alleviated disc degeneration, as evidenced by regularly arranged cartilage cells and fibrous annulus in the nucleus pulposus (Figure 5(c)).

We proposed a scoring system for the quantitative assessment of disc degeneration (Table 2). No significant differences in the histological scores were observed between the TP+NRF2 siRNA and degeneration groups (*P* > 0.05), but significant differences were detected between the TP group and the degeneration and TP+NRF2 siRNA groups. Both radiological and histological results provided evidence that TP treatment could delay the progression of IDD (Figure 6(d)).

### 3.8. Immunohistochemistry

Type II collagen and aggrecan expression was positive in NPCs treated with TP (yellow), but negative in TP+NRF2 siRNA and degeneration groups (blue) (Figures 6(e) and 6(f)). The expression of type X collagen, an indicator of nucleus pulposus degeneration, was markedly different in the three groups. These findings suggest that TP treatment alleviated the degeneration of NPCs compared to the saline degeneration and TP+NRF2 siRNA groups (Figure 6(g)).

SOD2, an indicator of oxidative stress, was strongly detected in the nucleus pulposus tissue in the degeneration and TP+NRF2 siRNA groups (yellow or brown), indicating an intense oxidative stress. However, staining of SOD2 was weaker in the TP group, suggesting that TP treatment suppressed oxidative stress in rats through the Keap1/Nrf2/ARE pathway, thereby delaying the degeneration of NPCs (Figure 6(h)).

## 4. Discussion

In the present study, we established an *in vitro* model for oxidative stress analysis in NPCs. Many studies have suggested that H_2_O_2_ could induce oxidative stress injury, inflammatory response, apoptosis, matrix reduction, and, finally, degeneration of NPCs [16–18]. Consistently, our study identified the degenerative role of H_2_O_2_ and established an *in vitro* model for oxidative stress analysis in NPCs. Data from the CCK-8 assay showed that H_2_O_2_ 100 *μ*M had no obvious effects on cell viability, whereas higher doses could suppress it. Thus, H_2_O_2_ at 100 *μ*M was chosen for further study. We found that H_2_O_2_ decreased the mRNA levels of type II collagen, aggrecan, and SOX-9 and increased type X collagen and MMP3. Moreover, H_2_O_2_ at 100 *μ*M dramatically suppressed the protein expression of type II collagen.

Type II collagen, an important component of the extracellular matrix of the nucleus pulposus, is mainly expressed in cartilage and cartilage-derived NPCs. Studies have shown that type II collagen is essential for the removal of the spinal cord and the formation of intervertebral discs [19]. Aggrecan, another important component of the extracellular matrix, has unique water-retaining and microstructural properties and serves a vital role in the intervertebral disc response to various loads [20]. The degradation and decreased synthesis of aggrecan could lead to a reduction in intervertebral disc function. SOX-9 is also a protective factor for intervertebral discs, which has the effect of regulating the expression of type II collagen [21]. When the intervertebral disc degenerates, the content of proteoglycan and type II collagen in the extracellular matrix decreases, with decreased water retention capacity and stress load capacity. The abovementioned three factors are all cartilage-specific genes. The reduction of type II collagen, aggrecan, and SOX-9 in NPCs is usually related to a severe disc degeneration [22]. Type X collagen indicates IDD, and it is a short-chain nonfibrous gel with a network structure. In adult intervertebral discs, type X collagen is highly expressed in the extracellular matrix of the intervertebral disc during the advanced stage of IDD [23, 24]. Type X collagen cross-links with type II collagen fibers, affecting the structure of the intervertebral disc and reducing its load strength. As a substance that destroys articular cartilage and intervertebral disc matrix, MMP3 is involved in disc degeneration, especially during the matrix degradation stage [25]. Therefore, type X collagen and MMP3 are highly expressed in degenerated NPCs. These results showed that 100 *μ*M H_2_O_2_ inhibited the expression of type II collagen, aggrecan, and SOX-9; increased type X collagen and MMP3; and accelerated the protein degradation of type II collagen, eventually causing degeneration of NPCs of intervertebral discs.

In the present study, we found that TP effectively reversed H_2_O_2_-induced downregulation of cartilage-specific genes. In addition, TP significantly reduced type X collagen and MMP3, indicators of IDD, which suggests that TP exhibits a protective role in NPCs. Western blotting results indicated that TP effectively increased the protein expression of type 2 collagen, whereas immunofluorescence staining showed that TP effectively reduced the expression of MMP3. This may be because of the fact that TP could increase the expression of type 2 collagen and suppress MMP3, thereby improving the microenvironment of NPCs and reducing type 2 collagen degradation. The protective effect of TP is concentration- and time-dependent at a certain concentration; that is, the protective effect of TP is more obvious at a larger dose in a longer intervention period. Furthermore, we explored the molecular mechanism by which TP protects degenerated NPCs. Previous studies have suggested that the prevention of DNA damage is associated with the anticancer role of tea and TP [12]. A recent study showed that TP attenuates oxidative stress induced by H_2_O_2_ and continuous darkness by regulating the Keap1/Nrf2 signaling pathway in HepG2 cells and mouse liver [26].

The Keap1/Nrf2/ARE pathway is critical for the defense against oxidative stress. Nrf2, a Cap'N'Collar family member, is widely expressed in human tissues and organs [27]. With a leucine zipper structure, Nrf2 activates ARE and initiates a variety of antioxidant reactions, thereby regulating the body's defense system against oxidative stress. Kelch-like ECH associating protein1 (Keap1) is the receptor of Nrf2 and affects its expression. Under physiological conditions, Nrf2 is located in the cytoplasm and binds to Keap1 in a resting state [28]. The Nrf2-Keap1 complex is rapidly degraded through the ubiquitin proteasome pathway. Upon oxidative stress stimulation, Nrf2 quickly disassociates from Keap1, translocates into the nucleus, binds to small Maf proteins to form heterodimers, and interacts with ARE in the target genes, thereby regulating the transcription and translation of antioxidant genes [29].

The activation of Nrf2 is regulated at multiple levels, mainly involving the interaction between Nrf2 and Keap1 as well as the mechanism mediating the stability of Nrf2. There are two mechanisms for the dissociation of Nrf2 from Keap1: the direct attack of nucleophiles or ROS and the indirect effect of phosphorylated Keap1.

In our study, there is evidence that H_2_O_2_-treated NPCs were attacked by ROS, leading to the disassociation of Nrf2 from Keap1. Elevated expression of Nrf2 increased the expression of ARE elements. TP exhibited a protective role in NPCs, increasing the expression of Nrf2 and downstream targets. These findings imply that TP may activate the Keap1/Nrf2/ARE pathway and protect NPCs against oxidative stress injury.

To test our hypothesis, we used DMF, the activator of the Keap1/Nrf2/ARE pathway, and observed that it exhibited a similar effect to that of TP. In addition, combined treatment with TP and DMF further increased the expression of Nrf2 and ARE. Collectively, these results suggest that TP activates the Keap1/Nrf2/ARE pathway to protect NPCs against H_2_O_2_.

The *in vivo* experiments also confirmed our hypothesis. The discs of rats were injected with TP (100 nM), TP+Nrf2 siRNA, and normal saline. Rats injected with normal saline exhibited obvious degenerated dark discs and low signal intensity. The likely causes are puncture-induced stress imbalance, oxidative stress injury, loss of proteoglycan, and type II collagen. In the normal saline group, the cartilage-like cells in the nucleus pulposus disappeared and the fibrous annulus was damaged. Treatment with TP at 100 nM greatly improved the degeneration of intervertebral discs in the first two weeks. Compared with the normal saline group, the T2-weighted image showed high signal intensity, indicating that the core components of the intervertebral discs were not destroyed. At week 4, the intervertebral disc signal of the rat also began to decrease, and the height of the intervertebral space narrowed, suggesting the degeneration of the nucleus pulposus. Histological analysis data, consistent with radiological results, suggested that the degeneration of intervertebral discs was alleviated in the TP group compared to that in the normal control *in vivo*. The addition of TP and Nrf2 siRNA produced similar effects on disc degeneration to the normal saline group, which was slower than in the control but faster than in the TP group. Immunohistochemical staining showed that TP significantly reduced the expression of oxidative stress factors in the tissues, whereas Nrf2 siRNA cotreatment and the degeneration groups showed a higher expression of SOD2. In summary, *in vivo* experiments showed that TP activated the Keap1/Nrf2/ARE pathway to exert its antioxidative stress function and ultimately delay the degeneration of intervertebral discs.

There were some limitations to this study. First, the long-term efficacy and safety of TP should be evaluated in the future. Second, this study mainly focused on the role of TP in signal transduction. The specific effect of TP on Nrf2 protein and the other key proteins in the pathway has not been determined. Besides, mitochondrial dysfunction is an important mechanism of oxidative stress damage in NPCs and should be further explored.

In summary, we have demonstrated that TP, a plant-derived natural antioxidant agent, plays an important role in activating the Keap1/Nrf2/ARE pathway and delaying disc degeneration. TP effectively increases the expression of NRF2 and its downstream targets, thereby inhibiting the degeneration of intervertebral discs. These findings reveal the protective function of TP via the Keap1/Nrf2/ARE pathway in intervertebral disc degeneration and deepen our understanding of IDD treatment and prevention.

## Figures and Tables

**Table 1:** Prime sequence.

iNOS	F: 5′-GATCA ATAAC CTGAA GCCCG-3′ R: 5′-GCCCT TTTTT GCTCC ATAGG-3′
NOX4	F: 5′-GAACCTCAACTGCAGCCTGATC-3′ R: 5′-CTTGTCAACAATCTTCTTCTTCTC-3′
SOD2	F: 5′-CTGGCCAAGGGAGATGTTAC-3′ R: 5′-AAAGACCCAAAGTCACGCTT-3′
LDH	F: 5′-TCATTCCTGCCATAGTCCA-3′ R: 5′-CAATTACACGAGTTACAGGTA-3′
T-AOC	F: 5′-CCAGGTGGCAGCCGACAATG-3′ R: 5′-ACGAAGACGAGGACGAGGATGG-3′
Collagen 2	F: 5′-TGAAGACCCAGACTGCCTCAA-3′ R: 5′-CGAGGTCAGCTGGGCAGAT-3′
Aggrecan	F: 5′-GGCATCGTGTTCCATTAC-3′ R: 5′-TCTCCATAGCAGCCTTCC-3′
SOX-9	F: 5′-ATCTGAAGAAGGAGAGCGAG-3′ R: 5′-TCAGAAGTCTCCAGAGCTTG-3′
Collagen X	F: 5′-AGTGTTTTACGCTGAACG-3′ R: 5′-TGCTCTCCTCTTACTGCT-3′
MMP-3	F: 5′-TATGGATCCCCCCCTGACTCCCCTGAG-3′ R: 5′-ATGGAATTCAGGTTCAAGCTTCCTGAGG-3′
NRF2	F: 5′-ACTCCGGCATTTCACTAAACACAAG-3′ R: 5′-CTGAGGCCAAGTAGTGTGTCTCCA-3′
KEAP1	F: 5′-CATCGGCATCGCCAACTTC-3′ R: 5′-ACCAGTTGGCAGTGGGACAG-3′
ARE	F: 5′-TCAGCAGCCACCACCTCCTAC-3′ R: 5′-AGTCACTACAGAGCCGCCATCC-3′

**Table 2:** Pfirrmann system.

Grade	Pfirrmann system			
	Structure	Signal	Disc height	Distinction between nucleus and anulus
I	Homogeneous	Bright hyperintense white signal intensity	Normal	Clear
II	Inhomogeneous	Hyperintense white signal	Normal	Clear, with or without horizontal gray bands
III	Inhomogeneous	Intermediate gray signal intensity	Normal or slightly decreased	Unclear
IV	Inhomogeneous	Hypointense dark gray signal intensity	Normal or moderately decreased	Lost
V	Inhomogeneous	Hypointense black signal intensity	Collapsed	Lost

**Table 3:** Grading for morphology.

		Morphology change under optical microscope	Grade
I	Annulus fibrosus (AF)	Normal texture and free of damage and distortion	1
		The damaged and distortion area is less than 30%	2
		The damaged and distortion area is more than 30%	3

II	Boundary between AF and NP	Normal	1
		Micro disrupted	2
		Medium or severe disrupted	3

III	NP cells	Normal cells with large amounts of vacuoles	1
		Cells and vacuoles decreased slightly	2
		Cells decreased moderately or severely without vacuoles	3

IV	NP matrix	Normal gel appearance	1
		Slightly congealed	2
		Moderate or severe condensation	3

## Data Availability

The datasets generated during and/or analyzed during the current study are available from the corresponding author on reasonable request.
